# A retrospective analysis among male and female infants EID results in Cross River State, Nigeria

**DOI:** 10.1186/1742-4690-9-S1-P43

**Published:** 2012-05-25

**Authors:** Onovo Amobi

**Affiliations:** 1 University Of Calabar, Nigeria; 2 Monitoring and Evaluation Officer at Pro-Health International, Calabar, Nigeria

## Background

Early definitive diagnosis of HIV infection in infants is critical to ensuring that HIV-infected infants receive appropriate and timely care and treatment. The purpose of this study is to investigate the possible determinant of EID test results among male and female infants in south-south region of Nigeria.

## Methods

A retrospective study was conducted in July, 2011 among male and female infants receiving PMTCT intervention for Early Infant Diagnosis in PHC's at four different LGA's of Cross River namely: Akamkpa, Calabar South, Odukpani and Biase respectively. Relevant data of the HIV infection status to male and female infants, whose samples were collected and diagnosed using PCR, was obtained from the National PMTCT-EID register. The data was analyzed using Cross-tabulation.

## Results

About 42.9% male infants and 57.1% female infant’s blood samples were collected using the DBS technology for diagnosis by PCR assay. The age distribution of the infants ranged from 2 - 11 months with the mean age of 5 months. EID samples (19.0%) diagnosed tested HIV positive and 81.0% tested HIV negative by PCR. 7.1% (Male) and 9.5% (Female) infants tested HIV positive by PCR. 35.7% (Male) and 47.6% (Female) infants tested HIV negative by PCR. There was a significant association between infant’s ages at 9 months, 10 months and 11 months with the EID test results (Standardized residual of 3.7, 1.9, and 2.6 respectively). The test of model of gender as predictor was statistically significant for female infants (Standardized residual of 1.6) and a Pearson chi-square which appeared statistically significant (P=0.006).

## Conclusion

From our study, there is a significant association between EID test results and specific ages of male and female infants with gender as a perfect predictor.

